# Case Report: Diagnostic and Therapeutic Challenges in Severe Mechanobullous Epidermolysis Bullosa Acquisita

**DOI:** 10.3389/fimmu.2022.883967

**Published:** 2022-04-07

**Authors:** Franziska Schauer, Alexander Nyström, Manfred Kunz, Stefanie Hübner, Sarah Scholl, Ioannis Athanasiou, Svenja Alter, Judith Fischer, Cristina Has, Dimitra Kiritsi

**Affiliations:** ^1^ Department of Dermatology, Medical Center - University of Freiburg, Faculty of Medicine, University of Freiburg, Freiburg, Germany; ^2^ Department of Dermatology, Venereology and Allergology, University Medical Center Leipzig, Leipzig, Germany; ^3^ Institute of Human Genetics, Medical Center - University of Freiburg, Faculty of Medicine, University of Freiburg, Freiburg, Germany

**Keywords:** rituximab, immunoglobulin, collagen VII, *COL7A1*, skin blistering, skin fragility

## Abstract

Collagen VII is the main constituent of the anchoring fibrils, important adhesive structures that attach the epidermis to the dermal extracellular matrix. Two disorders are caused by dysfunction of collagen VII, both characterized by skin and mucosa fragility, epidermolysis bullosa acquisita (EBA) and dystrophic epidermolysis bullosa (DEB). EBA and DEB share high clinical similarities with significant difference in patients’ age of onset and pathogenesis. Our patients presented with severe and recalcitrant mechanobullous EBA with characteristic DIF, IIF and ELISA diagnostics. But in both women recessive *COL7A1* variants were also found, in a monoallelic state. Collagen VII from EBA keratinocytes of our cases was significantly more vulnerable to proteolytic degradation than control keratinocytes, hinting that the heterozygous pathogenic variants were sufficient to destabilize the molecule *in vitro*. Thus, even if the amount and functionality of mutant and normal type VII collagen polypeptides is sufficient to assure dermal-epidermal adhesion in healthy individuals, the functionally-impaired proteins are probably more prone to development of autoantibodies against them. Our work suggests that testing for *COL7A1* genetic variants should be considered in patients with EBA, which either have a patient history hinting towards underlying dystrophic epidermolysis bullosa or pose therapeutic challenges.

## Introduction

Collagen VII is the main constituent of the anchoring fibrils, important adhesive structures that attach the epidermis to the dermal extracellular matrix (1). Structurally, it is formed by three identical alpha-chains consisting of a collagenous triple helix and the non-collagenous (NC) domains NC1 and NC2 (1). In the skin, it is produced by keratinocytes and papillary dermal fibroblasts and deposited at the dermo-epidermal junction zone (DEJZ) (2). In addition to the skin, collagen VII is also highly expressed in the oral cavity and oesophagus (2).

Two disorders are caused by dysfunction of collagen VII, both characterized by skin and mucosa fragility. In epidermolysis bullosa acquisita (EBA) reactivity of circulating and tissue bound IgG or IgA autoantibodies to collagen VII induces a structural detachment of the anchoring fibrils from the sublamina densa to the basement membrane zone (3). In contrast, the genetic disorder dystrophic epidermolysis bullosa (DEB) is caused by loss-of-function mutations in the gene encoding collagen VII (*COL7A1*) (2). These disorders share high clinical similarities. Patients of both disease groups develop chronic wounds in trauma-prone sites, have mucosal involvement associated with irreversible scarring of the affected areas and are prone to hair and nail loss. While in EBA the disease onset is usually in adulthood, in DEB skin blistering manifests primarily in neonatal age or early childhood (4).

Specifically, IgG autoantibodies in EBA have been shown to bind mainly to the NC1 domain of collagen VII; fewer patients show reactivity to either the collagenous domain or NC2 (5). The diagnosis is based on direct immunofluorescence (DIF) testing with u-serrated staining of IgG and/or IgA and C3 at the basement membrane zone, dermal staining of IgG and/or IgA in indirect immunofluorescence on salt split skin (ss-IIF) and detection of anti-collagen VII IgG autoantibodies by ELISA or immunoblot (3). Two commercial ELISA systems are available for standardized testing, with different sensitivity due to detection of autoantibodies against both the NC1/NC2 versus only NC1 domains (6).

A susceptibility locus, HLA-DR2, -DRB1*15:03, has been reported, accelerating the loss of tolerance by the affected person’s immune status (7). Furthermore, autoreactive collagen VII specific T-cells are required for induction of the disease, as reproduced in different animal models (8, 9). Treatment appears to be challenging especially in the mechanobullous form of the disease, since it has been reported to be resistant to oral immunosuppressants (5). Here we describe two women with severe mechanobullous recalcitrant EBA, summarizing the challenges in their diagnostic work up and treatment options.

## Case Description

### Case 1

**Figure 1:**
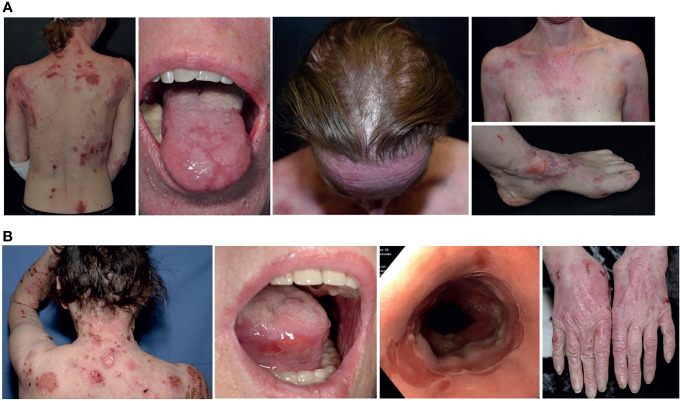
Clinical manifestations in both cases. **(A)** Clinical presentation of case 1 with blisters, erosions and scaring of her back, tongue, scarring alopecia, chest and nail dystrophy/loss presented at her left foot. **(B)** Case 2 shows similar lesions on her skin, mucous membranes (mouth and esophagus), as well as progressive scarring and milia on the backs of the hands. The finger nails are intact.

A 38-year old female was referred to our skin fragility center in Freiburg. The patient presented with a 10-year history of progressive skin fragility after her second pregnancy in 2008. She showed chronic blistering and scarring with severe involvement of skin, ocular, oral, esophageal and genital mucous membranes, nails and hair (**Figure 1A**). EBA or DEB was suspected for several years, since the autoimmune diagnostic was inconclusive. Past therapies initiated by different dermatologic specialists, including systemic glucocorticoids combined with chloroquine, azathioprine or mycophenolate mofetil, were unsuccessful. The detailed family history was unremarkable. The patient was otherwise healthy and had never taken any medication.

We started intensive topical treatment and 2 x 1000 mg of rituximab with subsequently monthly intravenous immunoglobin (IVIG) infusions. This regime had a stabilizing effect of her severely scarring skin condition with less blistering and fewer dressings, that allowed an occupational reintegration and active participation in daily life.

### Case 2

At admission, a 67-year old female reported that her first symptoms had occurred six months prior with blistering on the skin and mucous membranes, and dysphagia (**Figure 1B**). After diagnosing EBA, two cycles of high dose steroids combined with azathioprine (1.5 mg/kg) had to be stopped due to side effects (drug-induced liver injury). Another high dose steroid pulse was stopped due to complicating zoster oticus with acute and irreversible hearing loss. After admission to our department, treatments followed with long-term prednisolone at a dose between 70mg (1 mg/kg) tapered to 10mg, two cycles of rituximab (2 x 1000mg) and two cycles of intravenous immunoglobulins (IVIGs), combined with intensive topical treatment and wound care over 10 months. Due to constant recurrences, immune adsorption (IA) combined with prednisolone and mycophenolate mofetil was initiated, leading to partial clinical regression. Since the beginning of the first symptoms the patient experienced undulating fever up to 39.6°C, mostly connected to disease flaire-ups. Initially, no underlying neoplasia was identified, but around 1.5 years after diagnosis of EBA, a monoclonal gammopathy of unknown significance (MGUS) was identified, not requiring treatment. Rituximab cycles were poorly tolerated by our patient and IA led to septic thromboembolism requiring anticoagulation therapy in one course and bacterial septicaemia in two further treatments. Intravenously antibiotics had to be administrated to recover from these complications.

## Diagnostic Assessment

**Figure 2:**
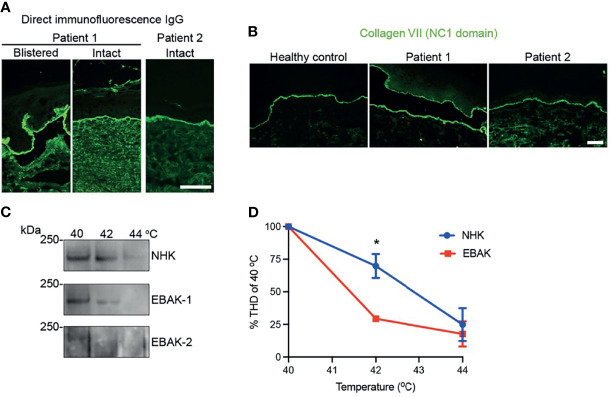
Results of the diagnostic assessment. **(A)** Direct immunofluorescence with linear IgG along the DEJZ and **(B)** immunofluorescence mapping of patient 1 and 2 showing regular staining of collagen VII compared to healthy control. Collagen VII of patient 1 stains at dermal and epidermal side within the blister, reminiscent of an older blister. Collagen VII of patient 2 shows wider depositions at DEJZ and interruptions due to microblistering. **(C)** Immunoblot of the trypsine-digested collagen VII shows degradation at lower temperatures in the patients’ keratinocyte lysates, as graphically depicted in **(D)**. * equals p: 0,0118 as tested with 2-WAY ANOVA.

In case 1, diagnostics revealed linear IgG deposits in DIF (**Figure 2A**), positive dermal stained ss-IIF, while ELISA diagnostics against the NC1 domain remained negative over years, hindering correct diagnosis (Euroimmun^®^ < 1 ratio; ratio > 1 considered positive). We added ELISA diagnostics with anti-collagen VII IgG recognizing both NC1/NC2 domains, which was then positive (MBL^®^ 45 U/ml; cut off >5 U/ml positive). Due to her severe clinical manifestations with scarring alopecia, enamel defects, nail dystrophy and esophageal webbing, a coexistent hereditary EB was suspected and an immunofluorescence mapping performed (10). It revealed a dermal blister with no obvious reduction in collagen VII (as shown by staining with an NC1 domain-directed antibody in **Figure 2B**). It is known that for collagen VII variants with slightly lower functionality, collagen VII expression at the DEJZ may not be affected, thus next generation sequencing EB gene panel analysis was performed as described before (11). The genetic variant c.7715G>T, p.Gly2572Val was found in the *COL7A1* gene in a heterozygous state. This variant is not reported in the Genome Aggregation Database v3.1.2, it substitutes a glycine in the Gly-X-Y repeat of the collagenous domain and is predicted to be pathogenic (Mutation Taster score 0.99; Polyphen2 score 1). Its pathogenic effect in a monoallelic state remains questionable, since it was also detected in the unaffected patient’s father. Glycine substitution in the distal collagenous domain affecting residues 2557 to 2590 have been reported to cause recessive DEB (12).

In patient 2, diagnosis of EBA was confirmed by histopathology, linear IgG deposits along the BMZ in the DIF (**Figure 2A**) and ss-IIF with dermal side staining of IgG. Anti-collagen VII IgG ELISA was positive with both systems tested (Euroimmun^®^ 5,13 ratio, ratio > 1 positive, MBL^®^ 112 U/, cut off >5 U/ml positive).

Due to the therapy-refractory course, immunofluorescence mapping was performed in parallel and showed expression of collagen VII comparable to control skin (**Figure 2B**). Mutation analysis of the *COL7A1* gene disclosed the heterozygous variant c.8227-1G>C, p.?, which affects the acceptor splice site in intron 110. Both c.8227-1G>C and c.8227-1G>A have been reported before to cause recessive dystrophic EB (13, 14). They may cause in frame skipping of exon 111.

We hypothesize that mutant polypeptides might interfere with triple helix formation to some extent and cause less stable collagen VII fibrils. To investigate this, we performed *in vitro* analyses of the thermal stability of the collagen triple helix. Keratinocytes from the patients skin (EBAK) were isolated and compared to control human keratinocytes (NHK) for the ability of collagen VII to withstand limited proteolytic digestion with trypsin, as described before (15). Proteolytic degradation of collagen VII would not be expected to be altered in EBA keratinocytes, without additional harmful functional effect from genetic variants. Interestingly, the collagen VII from EBAK was significantly more prone to proteolytic degradation than NHK in both cases, hinting that the heterozygous pathogenic variants were sufficient to destabilize the molecule *in vitro* (**Figures 2C, D**).

## Discussion

We describe two patients with a severe, therapeutically challenging EBA of the mechanobullous type. Both patients required a multidisciplinary management (otorhinolaryngology, gynaecology, gastroenterology, ophthalmology, hematology and infectiology), and systemic treatment encompassing immunomodulating and/or immunosuppressive therapies to prevent further fibrosis-related disease manifestations. Patient 1, who was already in a progressive stage at first contact, had the highest subjective benefit from rituximab 2 x 1000mg followed by IVIGs. Serological remission in patient 2 was only possible after combining multiple treatments over a period of 24 months. Notably, in patient 2 all cycles of rituximab were poorly tolerated, reminiscent of infusion reactions observed in lymphoma patients, owing to the systemic B-cell load (16). The role of MGUS and intermittent fever still remains elusive, since she did not meet the criteria required for diagnosing lymphoma. Around 20 EBA cases associated with a hematologic malignancy have been reported so far (17, 18), illustrating that repeated paraneoplastic screening may be considered, as is recommended for patients with laminin 332- associated mucous membrane pemphigoid (19). EBA following pregnancy is an extremely rare event and only reported in few cases (20). In general, the altered immune pattern with dominance of Th2 lymphocytes during pregnancy favours development of autoimmunity, while Th1 dominant diseases tend to improve (20). Whether stretching of the skin, causing damage to the extracellular matrix and connective tissue and thereby exposing maternal antigens plays a role, remains elusive.

Both *COL7A1* variants found in our patients are recessive and do not cause DEB in a monoallelic state. The amount and functionality of mutant and normal type VII collagen polypeptides is sufficient to assure dermal-epidermal adhesion. Similarly, few reports exist on autoimmunity and inflammatory imbalance in people with EB (21, 22), demonstrating that functionally-impaired proteins are probably more prone to development of autoantibodies against them. In sera of the studied EB patients autoantibodies against several components of the DEJZ, like collagen VII, BP180, BP230 and desmoglein 1 and 3 have been found. While negative direct and indirect immunofluorescence results suggest that circulating autoantibodies are most frequently non-pathogenic (23), some EB patients are at risk to develop an additional AIBD (24). Although appearing paradox on first sight, such observations are commonly observed in primary immunodeficiency disorders. There, genetic anomalies are associated with increased susceptibility for non- and infectious diseases, allergies and malignancies, but also enhanced autoimmunity (25). One can hypothesize, that structural impairment of the molecule, thereby facilitating tissue de-stabilization makes prone for autoimmunity due to an unstable protein, exposing otherwise hidden protein epitopes. Furthermore, it appears plausible that in such cases with an already slightly functionally impaired molecule, lower autoantibody titers suffice for a quick progression into a severe disease, with irreversible scaring and tissue fibrosis.

In EBA, 50% of the patients are described to be sero-negative (3). In addition, multiple cases have been reported, which are treatment-refractory, thus a detailed patient history is extremely valuable. Onychodystrophy in childhood or early enamel defects might hint towards a coexistent mild hereditary EB (26, 27). Guerra et al. also recommend a careful evaluation of EBA history and prompt mutation screening to unveil DEB variants (27).

In summary, testing for genetic variants in the *COL7A1* gene should be considered in patients with EBA, which either have a patient history hinting towards underlying dystrophic epidermolysis bullosa or pose therapeutic challenges.

## Patients’ Perspective

Both patients did adhere to the treatment proposed. Both women suffered from high disease activity, but were satisfied by the improvement of their clinical condition.

## Data Availability Statement

The original contributions presented in the study are included in the article/supplementary material. Further inquiries can be directed to the corresponding author.

## Ethics Statement

The studies involving human participants were reviewed and approved by Human Ethics Committee University of Freiburg (reference No.235/15). The patients/participants provided their written informed consent to participate in this study. Written informed consent was obtained from the individual(s) for the publication of any potentially identifiable images or data included in this article.

## Author Contributions

The idea for this research topic was brought up by FS and DK, who also performed data acquisition and sorting. Structural analyses were done by FS, DK, CH, JF, AN, IA, SS, and SH. The manuscript was drafted and designed by FS and DK. MK provided patient data and revised the manuscript. IA performed the trypsin digestion assays, KT the serological analyses. The mutation analysis data from both patients were evaluated by CH and JF. All authors have read and approved the final manuscript.

## Funding

FS and DK were funded by the Berta-Ottenstein-Programme for Advanced Clinician Scientists, Faculty of Medicine, University of Freiburg. DK is also funded by the German Research Foundation (DFG) through SFB1160 project B03, SFB-1479 – Project ID: 441891347 and KI1795/2-1 and the Fritz Thyssen Foundation.

## Conflict of Interest

The authors declare that the research was conducted in the absence of any commercial or financial relationships that could be construed as a potential conflict of interest.

## Publisher’s Note

All claims expressed in this article are solely those of the authors and do not necessarily represent those of their affiliated organizations, or those of the publisher, the editors and the reviewers. Any product that may be evaluated in this article, or claim that may be made by its manufacturer, is not guaranteed or endorsed by the publisher.
